# Altered neural substrates within cognitive networks of postpartum women during working memory process and resting-state

**DOI:** 10.1038/s41598-020-66058-x

**Published:** 2020-06-04

**Authors:** Yunjin Bak, Yoonjin Nah, Sanghoon Han, Seung-Koo Lee, Na-Young Shin

**Affiliations:** 1 Department of Radiology, College of Medicine, The Catholic University of Korea, Seoul, 06591 Korea; 2 Department of Psychology, Yonsei University, Seoul, 03722 Korea; 3 Department of Radiology, Yonsei University College of Medicine, Seoul, 03722 Korea

**Keywords:** Cognitive control, Reproductive signs and symptoms

## Abstract

Postpartum working memory decline has been investigated mostly with neuropsychological tests, but neural evidence is almost unknown. Here we investigated task-related neural alterations during working memory task (n-back) and intrinsic alterations during resting-state (rs) in postpartum women using functional MRI (fMRI). Behaviorally, postpartum women showed comparable working memory performances to the controls although there was a tendency of prolonged response time. fMRI analysis results showed hyper-activation in regions belong to the task positive network (TPN) during the task and hypo-rsfMRI values in the default mode network (DMN) regions during rest in postpartum women. Based on these results, we performed network connectivity analysis using nodes of the TPN and DMN. As a result, the DMN showed a tendency of decreased connectivity in postpartum women during the working memory process compared to the controls. Our results suggest that postpartum women might have functional alterations in the DMN, and that hyper-activation in the TPN during a task might be a compensatory mechanism to maintain working memory performance in postpartum women.

## Introduction

A large number of postpartum women frequently complain of memory decline that disturbs their daily activities^1^. Thus, several studies have attempted to find empirical evidence of postpartum memory decline using various neuropsychological tests^2–6^. Working memory has been examined in most of these studies, but the results have been very inconsistent, with some studies reporting significant working memory impairment^4,7^, while others failing to find any deficiency^2,8^. Although one meta-analysis tried to give a possible explanation for this disparity by showing that postpartum women are selectively impaired in tasks demanding relatively high cognitive loads on the executive component of working memory than the storage component of working memory^7^, the reason behind these conflicting results is still under debate. As working memory processes play a fundamental role in higher cognitive functions^9^, which affect the lives of postpartum women not only in their caregiving but also in their work, it is important to accurately identify changes occurring to working memory during the postpartum period.

Neural response can be more sensitive than behavioral neurocognitive tests^10,11^, but the underlying neural changes for postpartum working memory remain poorly understood. Previous studies demonstrated that the working memory process mainly involves a set of regions such as the dorsolateral prefrontal cortex (dlPFC) and inferior parietal lobule described as the fronto-parietal network or task-positive network (TPN)^12,13^. On the other hand, there are regions anti-correlated with the TPN, which are known as the default mode network (DMN), and the relationship between these two networks is reported to play an important role in cognitive processing^12^. Although the DMN was mostly characterized in task-free context and not considered to be involved in task processing, studies were eventually able to show that DMN connectivity during working memory tasks and even resting-state activation is significantly correlated with working memory task performance^14–16^. Therefore, considering the importance of the TPN and DMN on working memory, we hypothesized that these two networks would reveal neural changes in postpartum women.

One of the most distinct characteristics of the postpartum period is the dramatic change in hormone levels after delivery: a sharp decrease of estrogen and progesterone levels after gradual increase during pregnancy^17^. Estradiol levels have been reported to have a high correlation with working memory ability in healthy young women^18,19^ and postmenopausal women^20,21^. Given the association, we can postulate that low estradiol levels in postpartum women might affect their working memory change. However, little information is available to define the association between estradiol level and working memory performance in postpartum women^6^. Thus, in this study, we also tried to examine the association between working memory ability and estradiol level.

In the present study, we aimed to investigate neural alterations of activity and connectivity in postpartum women during the working memory process. fMRI activation patterns during an n-back task were analyzed and compared between the postpartum group and controls who had no history of pregnancy. In order to examine intrinsically altered brain activity and connectivity which have not been well identified in postpartum women, we conducted three resting-state fMRI (rsfMRI) analyses in a data-driven manner. We expected there would be some consistently altered areas present, especially in the working memory network regions for all three analyses. Using network nodes derived from the resting-state data, a network connectivity analysis was conducted within the nodes of the DMN and TPN during an n-back task, and the relationships between fMRI values, clinical data and working memory performance were assessed.

## Results

### Participant characteristics

The participants’ demographic and clinical data are given in Table 1. The postpartum group reported more nocturnal awakenings than the control group (*P* < 0.001). They also complained of subjective cognitive decline more often compared to the control group (higher Cognitive Failure Questionnaire (CFQ) scores; *P* = 0.008), and had more severe depressive symptoms than the control group (high Beck Depression Inventory (BDI) scores; *P* = 0.043). The serum estradiol level of the postpartum group was more than two times lower than that of the control group (*P* = 0.007). Among the neuropsychological test results, the postpartum group showed poor performance only in the Digit Span Sequencing test which was designed to measure working memory performance (*P* = 0.031).

**Table 1:** Demographic and clinical characteristics.

	Control group (n = 27)	Postpartum group (n = 24)	*P* value
**Demographic characteristics**			
Age (y)	29.4 ± 4.3	31.0 ± 3.0	0.111
Duration of education (y)	16.0 (16.0–19.0)	16.0 (16.0–19.0)	0.279
Married	6 (22.2%)	24 (100%)	
Normal delivery/C-sec	N/A	17/7	N/A
Number of children	N/A	1 (1.0 – 2.0)	
Breast feeding	N/A	14 (58.33%)	N/A
Interval between MRI scan and delivery date (d)	N/A	103.3 ± 16.4	N/A
**Self-report questionnaires**			
Number of awakenings	0.0 (0.0–0.0)	1.75 (0.3–2.0)	<0.001
Total sleep time per day (h)	6.0 (6.0–7.0)	7.0 (6.0–8.5)	0.099
Lack of sleep	5.0 (2.0–7.0)	4.5 (3.0–6.0)	0.863
EPDS	5.0 (3.8–8.0)	5.5 (2.0 – 11.25)	0.882
BDI	5.0 (3.0–8.0)	8 (5. 5 – 10.0)	0.043
CFQ	26.8 ± 9.5	35.5 ± 12.8	0.008
Estradiol assay (pg/mL)	89 (52.0–201.0)	40.5 (25.0–66.5)	0.007
**Neuropsychological test**			
COWAT (Animal)	29.0 ± 4.5	20.1 ± 5.5	0.550
15-item K-BNT	14.0 (14.0–15.0)	14.0 (14.0–15.0)	0.621
Digit span forward	15.0 (14.0–16.0)	14.0 (13.0–15.0)	0.073
Digit span backward	13.0 (11.0–14.0)	12.5 (9.0–14.0)	0.321
Digit span sequencing	9.4 ± 2.1	8.0 ± 2.5	0.031*
Word List Memory	26.0 (24.0–27.5)	26.0 (24.0–27.0)	0.563
Word List Recall	90.0 (86.8–100.0)	100.0 (90.0–100.0)	0.068
Word List Recognition	10.0 (10.0–10.0)	10.0 (10.0–10.0)	0.342
TMT_A	20.5 (19.0–25.0)	20.5 (18.0–28.0)	0.778
TMT_B	51.5 (42.8–80.8)	51.0 (38.5–61.0)	0.260

Normally distributed data are expressed as means ± standard deviations; otherwise, data are expressed as medians with the interquartile range in parentheses. Abbreviations: BDI, Beck Depression Inventory; CFQ, Cognitive Failure Questionnaire; COWAT, Controlled Oral Word Association test; EPDS, Edinburgh Postnatal Depression Scale; 15-item K-BNT, 15-item Korean version of the modified Boston Naming Test; and TMT, Trail Making Test.

### N-back task performance

Repeated-measures ANOVA was conducted to investigate group differences (group: postpartum group vs. control group) in task performance for the n-back task with varying cognitive loads (task: 0-back vs. 1-back vs. 2-back).

In terms of main effect of group, there was no significant difference in both accuracy and reaction time (RT) between the two groups. The postpartum group only showed a tendency of overall increased RT irrespective of conditions compared to the control group (*F*(1, 49) = 3.63, *P* = 0.063; Table 2). For the main effect of task, significant differences were observed in both accuracy and RT (Wilks’ Lambda = 0.53, *F*(2,48) = 21, *P* < 0.001 and Wilks’ Lambda = 0.36, *F*(2,48) = 43, *P* < 0.001, respectively). Post-hoc t-test analyses showed slower RT and decreased accuracy with increasing working memory load in the [2-back – 0-back] contrast (RT: *t*(50) = 5.43, *P* < 0.001; Accuracy: *t*(50) = −3.48, *P* = 0.001) and the [2-back – 1-back] contrast (RT: *t*(50) = 8.84, *P* < 0.001; Accuracy: *t*(50) = −6.39, *P* < 0.001) in all participants. However, this pattern was not observed with the [1-back – 0-back] contrast. Considering that increased cognitive load usually entails impaired performance, we assumed that an appropriate cognitive load effect was not reflected in the [1-back – 0-back] contrast. Therefore, only the [2-back – 0-back] contrast and the [2-back – 1-back] contrast were included in subsequent fMRI analyses.

**Table 2:** Behavioral results of n-back task.

	Control group (n = 27)	Postpartum group (n = 24)	*P* value
**RT (ms)**			
0-back	476.7 ± 49.5	496.6 ± 50.7	0.163
1-back	436.2 ± 47.7	464.0 ± 58.9	0.069
2-back	533.3 ± 83.0	560.8 ± 72.8	0.218
overall*	482.2 ± 44.9	507.1 ± 49.0	0.063
**Accuracy**			
0-back	100.0 (91.7–100.0)	(100.0 (100.0–100.0)	0.348
1-back	100.0 (100.0–100.0)	(100.0 (100.0–100.0)	0.770
2-back	91.7 (75.0–100.0)	91.7 (83.3–91.7)	0.415
overall	94.4 (86.1–100.0)	94.4 (88.9–97.2)	0.909

Normally distributed data are expressed as mean ± standard deviation; otherwise, data are expressed as medians with the interquartile range in parentheses. Abbreviations: RT, Response time.

There was no interaction effect between group and task. Even after BDI, CFQ, and number of awakenings were included as covariates because they showed significant group differences, there were still no interaction effects between group and task on accuracy and RT of the n-back task.

There were also no significant correlations between performance on the Digit Span Sequencing test with the accuracy and RT of the n-back task.

### fMRI results

#### n-back task-related activation analysis

**Figure 1:**
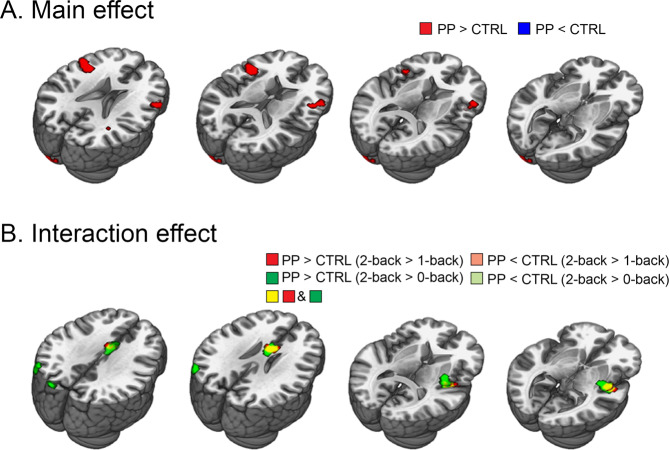
n-back task-based fMRI activation analysis results. (**A**) Main effects revealed by 2 (group: PP vs. CTRL) x 3 (task: 0-back vs. 1-back vs. 2-back) full factorial analysis. There is no significant voxel in the [PP < CTRL] contrast. (**B**) Interaction effects revealed by 2 (group: PP vs. CTRL) x 3 (task: 0-back vs. 1-back vs. 2-back) full factorial analysis. There is no significant voxel in the [PP < CTRL (2-back > 1-back)] contrast and the [PP < CTRL (2-back > 0-back)] contrast. Abbreviations: CTRL, control group; PP, postpartum group.

We first investigated the main effect of group on n-back task-related brain activation. Compared to the controls, the postpartum group showed increased activation in the bilateral dlPFC. There were no significant clusters showing decreased activation relative to the control group (Fig. 1A and Table 3).

**Table 3:** n-back task-based fMRI activation analysis results.

Contrast	Connected region	Side	MNI coordinates			Maximum *t*	No. of voxels	*P* value
			x	y	z			
***Main effect***								
PP > CTRL	Dorsal lateral prefrontal gyrus	left	−48	11	19	3.6	132	<0.001
	Dorsal lateral prefrontal gyrus	right	60	26	25	3.28	72	0.001
***Interaction effect***								
PP (2-back - 1-back) > CTRL (2-back - 1-back)	Insula/Putamen	right	45	−7	−5	3.39	86	<0.001
	Anterior cingulate cortex	left	0	8	22	2.87	37	0.002
PP (2-back - 1-back) <CTRL (2-back - 1-back)	Insula/Putamen	left	45	−7	−5	3.62	104	<0.001
	Anterior cingulate cortex	left	0	8	22	3.56	65	<0.001

Regions which showed significant differences in brain activation on 2 (group: PP vs. CTRL) x 3 (task: 0-back vs. 1-back vs. 2-back) full factorial analysis. Abbreviations: CTRL, control group; PP, postpartum group.

Next, the interaction effect between group and task was inspected. With increasing cognitive demands on the [2-back – 0-back] contrast, the postpartum group showed increased activation in the anterior cingulate cortex (ACC), insula/putamen and middle occipital gyrus compared to the control group. For the [2-back – 1-back] contrast, the ACC and insula/putamen showed identical activity patterns to the [2-back – 0-back] contrast, with the peak coordinates of each cluster even being the same, but the middle occipital gyrus did not reach statistical significance in this contrast (Fig. 1B and Table 3). In addition, even when controlling for CFQ and BDI, and number of awakening which all showed significant group difference, identical regions including the ACC and insula/putamen still showed significant interaction effect (Supplementary information Fig. S1). These results showed that neural regions known as the TPN, which includes the dlPFC, ACC and insula, show increased activation in the postpartum group relative to the control group even after controlling the effect of the depressive and subjective cognitive decline symptoms as well as sleep quality.

#### Resting-state analysis

**Figure 2:**
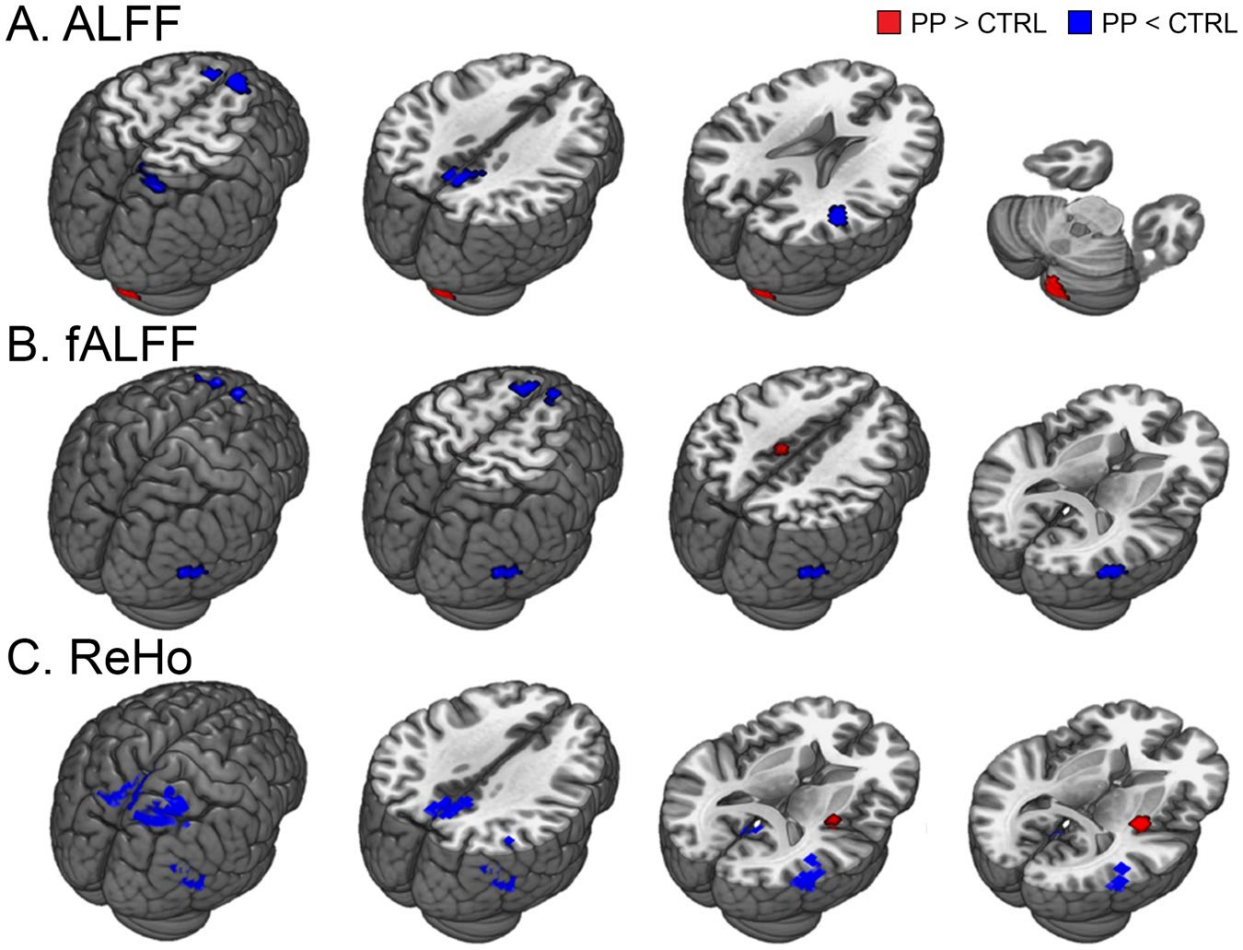
Resting-state fMRI analysis results. Regions showing significant group differences in ALFF, fALFF and ReHo values on the two-sample t-test. Abbreviations: CTRL, control group; PP, postpartum group.

Three resting-state analyses assessed the amplitude of low frequency fluctuations (ALFF), fractional ALFF (fALFF), and regional homogeneity (ReHo), which reflect the amplitude and connectivity of regional spontaneous neural signals, and consistently showed those values to be decreased in the DMN regions (Fig. 2 and Table 4). In the ALFF analysis, a group comparison between the postpartum and control groups revealed reduced ALFF in the medial prefrontal cortex (mPFC) and right temporoparietal junction (rTPJ) of the postpartum group, which are the main structures of the DMN (Fig. 2A). Increased ALFF values were observed in the cerebellum, occipital gyrus and precuneus of the postpartum group. The fALFF analysis also demonstrated decreased fALFF in the postpartum group in the DMN areas, including the mPFC, rTPJ and inferior temporal gyrus (Fig. 2B). Furthermore, the resulting mPFC cluster (43 voxels) overlapped with the ALFF results. The ReHo analysis showed decreased ReHo values in the postpartum group in the posterior cingulate cortex (PCC) and rTPJ which also belong to the DMN (Fig. 2C). The PCC (22 voxels), rTPJ (43 voxels), and right superior parietal region (15 voxels) clusters in ReHo overlapped with the ALFF clusters. In addition, the middle temporal gyrus cluster in ReHo also overlapped with the corresponding cluster of fALFF (10 voxels). The cerebellum and insula/putamen showed increased ReHo values in the postpartum group.

**Table 4:** Resting-state fMRI analysis results.

Contrast	Connected region	Side	MNI coordinates			Maximum *t*	No. of voxels	*P* value
			x	y	z			
***ALFF***								
PP < CTRL	Angular gyrus	right	42	−45	24	4.65	53	<0.001
	Precuneus	right	3	−57	42	4.25	34	<0.001
	Dorsal medial frontal gyrus	left	−9	24	60	4.11	30	<0.001
	Dorsal medial frontal gyrus	right	9	30	57	4.01	45	<0.001
	Orbitoinferior frontal gyrus	right	36	51	−12	3.79	45	<0.001
	Superior parietal gyrus	right	18	−66	63	3.77	37	<0.001
PP > CTRL	Cerebellum	right	21	−60	−51	3.16	27	0.001
***fALFF***								
PP < CTRL	Superior medial frontal gyrus	left	−9	27	66	4.63	60	<0.001
	Middle temporal gyrus	right	57	−69	15	4.02	28	<0.001
	Dorsal medial frontal gyrus	right	9	30	66	3.92	31	<0.001
PP > CTRL	Paracentral lobule	right	−9	−24	45	3.8	43	<0.002
***ReHo***								
PP < CTRL	Superior temporal gyrus	right	45	−48	27	5.55	68	<0.001
	Post cingulate cortex	right	0	−51	18	5.01	41	<0.001
	Precuneus	right	3	−60	42	4.39	60	<0.001
	Middle temporal gyrus	right	54	−63	15	4.26	42	<0.001
	Superior parietal gyrus	left	33	−54	69	3.94	129	<0.001
PP > CTRL	Putamen	right	33	−15	6	4.93	40	<0.001
	Cerebellum	left	−21	−60	−57	4.61	60	<0.001
	Cerebellum	left	21	−63	−48	4.47	51	<0.001
	Cerebellum	left	−18	−51	−27	4.33	89	<0.001
	Cerebellum	right	−33	−45	−45	4.09	35	<0.001
	Precentral gyrus	left	−33	−3	54	3.88	25	<0.001

Regions which showed significant group differences in ALFF, fALFF and ReHo values on two-sample t-test. Abbreviations: CTRL, control group; PP, postpartum group.

#### n-back task-related network connectivity analysis

**Figure 3:**
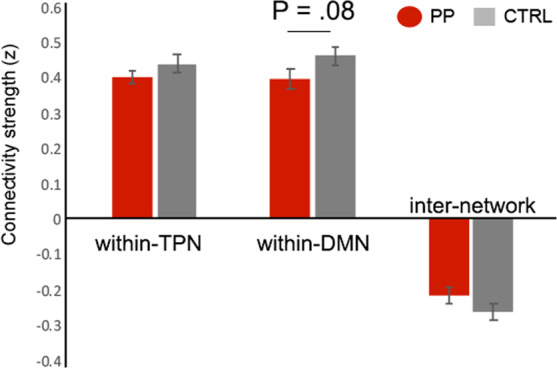
Group differences in the within-TPN, within-DMN and DMN-TPN inter-network connectivity strengths during the n-back task. Abbreviations: CTRL, control group; PP, postpartum group.

Connectivity analysis at the network level during the n-back task revealed significant positive correlation for within-DMN and within-TPN connectivity in both the postpartum and control groups, while significant anti-correlation was observed between the two networks in both groups. Group comparisons of within-DMN, within-TPN and DMN-TPN connectivity only yielded a trend of reduced within-DMN connectivity in the postpartum group (*P* = 0.080). There was no significant difference in within-TPN connectivity and DMN-TPN inter-network coupling between the postpartum group and the control group (Fig. 3).

#### Correlation analysis between estradiol level, working memory performance and network connectivity

**Figure 4:**
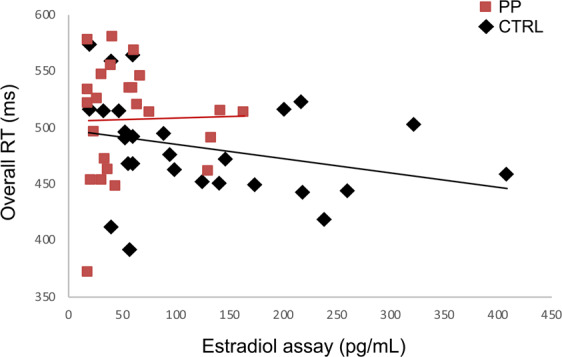
Correlation analysis between estradiol level and overall RT. Abbreviations: CTRL, control group; PP, postpartum group.

A significant negative correlation was found between the serum estradiol level and overall RT (Spearman’s *rho* = −.304, *P* = 0.030). A negative correlation was significant in the control group (Spearman’s *rho* = −0.402, *P* = 0.038), but not in the postpartum group (Spearman’s *rho* = 0.007, *P* = 0.974; Fig. 4). In order to check if the interaction between estradiol level and group significantly affected overall RT, we used the glm R function including group, estradiol level, and their interaction term group*estradiol level in the model. Group (reference = control group; *β* = 4.700; standard error [SE] = 20.979; *P* = 0.824), estradiol level (*β* = −0.153; SE = 0.088; *P* = 0.088), and interaction effect (*β* = 0.158; SE = 0.234; *P* = 0.503) did not have any significant effect on the overall RT.

The estradiol levels also did not show any significant correlation with the DMN-TPN inter-network, within-DMN, and within-TPN connectivities.

#### Voxel-based morphometry analysis

**Figure 5:**
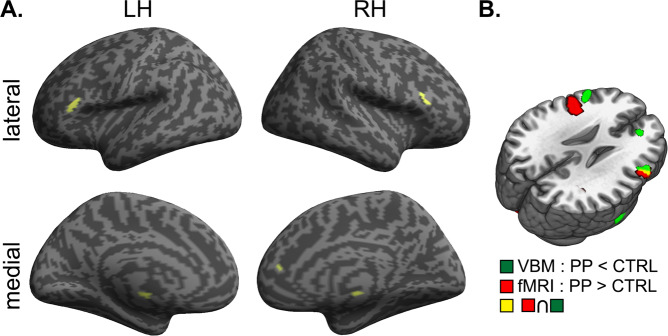
VBM analysis result. (**A**) Regions showing significant atrophy in the postpartum group compared to the control group. (**B**) Overlapping regions between cortical atrophy and increased activation during n-back test in the postpartum group. Abbreviations: CTRL, control group; PP, postpartum group.

The postpartum group showed reduced gray matter volume in the bilateral dlPFC, right ACC, right middle temporal gyrus, and left pallidum, which overlapped with TPN regions hyper-activated during the n-back task (uncorrected P < 0.001, k = 100). Gray matter volume did not decrease more in the control group compared with the postpartum group (Fig. 5).

## Discussion

In this study, we investigated neural alterations of postpartum women during the working memory process and resting-state. Although postpartum women showed comparable working memory performance to controls, they showed different neural activation. The postpartum group showed hyper-activity in TPN regions with increasing cognitive load during the working memory process, while showing hypo-activity in DMN regions during resting-state compared to the control group. A subsequent network connectivity analysis also implied possible alterations in the DMN during working memory task. Therefore, postpartum women might have functional alterations in DMN regions, and TPN regions might be hyper-activated to maintain working memory performance in postpartum women.

As some previous studies reported^2,8^, the postpartum group did not show significant difference in performances for the n-back task compared to the controls in our study. However, the postpartum group showed a tendency of overall prolonged RT compared to the controls. As RT represents the speed of information processing, the prolonged RT of the postpartum group may reflect decreased speed in information processing. Decreased processing speed has been consistently reported in postpartum women^4–6,8,22^. Although our postpartum group did not show significant reduction in processing speed during the task, slower processing speeds could be worse or more prominent in their daily life. In studies that investigated pregnant cognitive change, significant memory deficit was found in field tasks but not in laboratory tasks^23,24^. It appears that pregnancy-related memory decline may be better detected when women face the actual competing demands of everyday life. RT was also prolonged as working memory load increased in our study. Therefore, we postulated that postpartum women felt the working memory task was more difficult than the controls did, with somewhat decreased ability to perform the task.

Although there was no significant difference in working memory performance, the postpartum group showed more activation in TPN than the control group. The hyper-activation in TPN regions during cognitive tasks has been reported in patients with mild cognitive impairment who have shown comparable performance to controls, but not in those with significant cognitive decline in previous studies^25–27^. The authors of these past studies suggested this finding as a compensatory process or inefficiency. Therefore, the hyper-activation of TPN regions in our postpartum group could also be interpreted in the same manner. In addition, the postpartum group showed reduced gray matter volume in the bilateral dlPFC, ACC, and pallidum which largely belong to the TPN in our study. Specifically, the dlPFC which showed gray matter volume loss partly overlapped with the areas hyper-activated during the n-back task in the postpartum group compared to the controls. This dlPFC volume loss was also reported with increased neural activity in corresponding areas in a previous study on postpartum women^28^. These findings suggest that the dlPFC can be hyper-activated to make up for volume loss, and corroborates the existence of a compensatory mechanism in the TPN of postpartum women.

The postpartum group consistently showed DMN alterations in three different analyses during resting-state when no tasks were performed. It is presumed that resting-state brain organization reflects more fundamental and intrinsic neural properties than transient task-evoked neural signals^29^. Moreover, a recent study found that the organization of the resting-state network shapes neural activation for cognitive tasks^30^. This raises the possibility that DMN disruption might be an intrinsic change occurring during the postpartum period and that this change can lead to alterations in neural activation patterns during the task as well as behavioral performance. According to the previously-mentioned recent study^28^, prolonged atrophy in regions which overlap with the DMN was found in postpartum women and lasted for more than two years after birth. Furthermore, those atrophic areas showed increased neural activity when the postpartum women saw pictures of their own babies compared to other infants, which suggests that the DMN is involved in the behavior which facilitates motherhood. Accordingly, underlying functional and structural alterations might be attributable to specific behavioral patterns of postpartum women, and we suggest the DMN as a core region for these alterations.

Although the influence of estradiol on the specific types of cognitive abilities including working memory has been consistently reported^18–21^, the exact mechanism has not been well established. According to some previous studies, estrogen might play a role as a mediator, inducing neural changes in several brain areas^31,32^. In those studies, estrogen level was correlated with neural activation in the insula and postcentral gyrus in young healthy women^31^ and in the prefrontal area in postmenopausal women^32^ during working memory tasks. However, we could not find a significant relationship between estrogen and the activity as well as the network connectivity of DMN and TPN in our study. As the effect of estrogen on neural activation and subsequently, on cognitive function has not been well studied in postpartum women, associations between estrogen, neural index from activity and connectivity, and cognition have to be further elucidated in future studies.

In contrast to the fMRI values, prolonged RT was significantly correlated with lower serum levels of estradiol in our study, supporting the positive effect of estradiol on working memory function. However, the association was significant in the control group, while significance was not found in the postpartum group. The relatively narrow range of the low serum levels of estradiol in the postpartum group could be a possible explanation, at least in part, for the differential results.

There are some limitations of this study. First, since we mainly focused on the DMN and TPN, possible alterations in other cognitive networks during the postpartum period were not examined. Future studies may consider expanding the investigation to other networks, which can supplement our results. Second, motivation could be a significant factor affecting the attention on irrelevant stimuli, but was not assessed and controlled for in this study. Since women during the postpartum period usually engage in many new tasks, low motivation might lead to distraction by irrelevant stimuli. Thus, motivation itself can be a factor and needs to be corrected for in future studies. Third, the number of participants were small in our study, resulting in an inherent limitation when generalizing our results. However, this study was exploratory research that was focused on seeking neural substrates relevant to postpartum working memory change, which has not been investigated before, and its findings can help facilitate future studies in this research field.

## Conclusion

This study examined neural alterations during working memory process and resting state in postpartum women. Even though postpartum women showed comparable working memory performance, there was decreased activity and connectivity in the DMN regions during resting state as well as the working memory process in postpartum women. Postpartum women also showed hyper-activation in the TPN regions during the working memory task, which might be due to a compensation mechanism to maintain working memory performance. Since neural correlates of postpartum cognitive change have not yet been sufficiently studied, our results might provide potential neural targets associated with postpartum working memory changes for future studies.

## Methods

### Participants

Twenty-five females in the 2^nd^ to 4^th^ month after parturition (the postpartum group) with normal pregnancies, uncomplicated term vaginal or Caesarean deliveries, and healthy babies were recruited. Thirty-two age-matched, naturally cycling women who had never experienced pregnancy participated as controls in the experiment. All participants were right-handed and native Koreans with normal or corrected-to-normal vision. Exclusion criteria included treatment with hormonal preparations, treatment with psychotropic drugs, medical, psychiatric, or neurological comorbidities that might be associated with cognitive dysfunction, and history of head trauma that resulted in loss of consciousness or concussion. This prospective study was approved by the Institutional Review Board of Severence Hospital, ant all participants signed a written informed consent document after receiving a full explanation of the study protocol. The experiment was carried out in accordance with the latest version of the Declaration of Helsinki. In data analysis, five participants of the control group and one participant of the postpartum group were excluded for the following reasons: head movement exceeding the priori maximum of 3 mm (n = 2), severe signal loss in the parietal lobe (n = 2), and an age 1.5 standard deviations below the mean age (n = 1).

### Experimental overview

The present experiment consisted of three sessions. In the pre-scan session, participants responded to questionnaires for demographic and clinical characteristics, and blood samples were collected for the estradiol assay. During the fMRI scanning session, participants underwent resting-state scanning and performed remember-know encoding task, n-back task, prospective memory task, remember-know retrieval task and empathy task. Then, 3 dimensional (3D) T1-weighted structural imaging was acquired. After scanning was complete, the neuropsychological test battery was conducted to assess cognitive function. We only reported the n-back task and resting-state data in this present study.

### Self-report questionnaires

Participants were asked to fill out questionnaires about demographic and clinical characteristics, number of nocturnal awakenings, total sleep time per day, and a 10-point scale to rate sleep deprivation in which 0 indicated no sleep deprivation and 10 severe deprivation. The questionnaires also included the BDI^33^ and the Edinburgh Postnatal Depression Scale (EPDS)^34^ to assess depressive symptoms, and the CFQ^35^ to assess subjective cognitive decline.

### Estradiol assays

All blood samples were obtained by venipuncture from the antecubital space, and serum estradiol levels were estimated by an enzyme chemiluminescent immunoassay using a UniCel DxI 800 automated analyzer (Beckman Coulter, Inc, Fullerton, CA). The minimum sensitivity and range for estradiol levels was 20-4800 pg/mL.

### Neuropsychological tests

The neuropsychological test battery included the forward, backward, and sequencing Digit Span subsets of the Korean version of the Wechsler Adult Intelligence Scale (K-WAIS) to evaluate working memory^36^, the TMT part A to evaluate speed of information processing^37^, the semantic Controlled Oral Word Association test (COWAT) and Trail Making Test (TMT) part B to evaluate executive function/verbal fluency^37^, the 15-item Korean version of the modified Boston Naming Test (K-BNT) to evaluate language function, the Word List Memory to evaluate verbal learning, and the Word List Recall and Recognition to evaluate verbal learning/memory function.

### Working memory paradigm: the n-back task

The current study employed a letter variant of the n-back task paradigm in which three n-back blocks ranging from 0-back to 2-back interleaved with four fixation blocks (F). The n-back blocks and fixation blocks were presented in fixed order ((F, 0-back, 1-back, 2-back) x 3, F), and each block lasted for 40 s including 3 s for the instruction page at the beginning of the block. In the 0-back block, participants were required to press a button with their right index finger on the appearance of the letter Z in a string of letters. For the 1-back and 2-back blocks, participants had to respond with the same button whenever the current letter was identical to the letter right before (1-back), or the letter two spaces before (2-back). Each n-back block was composed of four target trials and fifteen non-target trials. Letters in white font were presented serially in the center of the black screen for 1 s with an inter-trial interval of 1 s. During the fixation blocks, a central fixation cross was displayed, and participants were instructed to stare at the cross and relax. Accuracy was computed by calculating the percentage of correct answers to total answers. RT was measured in milliseconds from the presentation of each stimulus until participants pressed the button. Accuracy and RT were computed for each task condition and also for the overall task.

### Image acquisition

All scans were acquired by using a 3 T MR imaging unit (Discovery MR750; GE Healthcare, Milwaukee, WI) with an 8-channel head coil. Functional images were acquired with the following parameters for the gradient-echo single-shot echoplanar imaging (GE-SS-EPI) sequence: TR = 2000 ms, TE = 30 ms, FOV = 240 × 240 mm^2^, voxel size = 3.75 × 3.75 × 4.0 mm^3^, flip angle = 90°, 33 axial slices tilted 30° from the AC–PC plane, no gap, interleaved. rsfMRI data were collected and followed by five functional runs of volume acquisition. During the resting-state data scan which lasted for 6 min 48 sec, participants were instructed to close their eyes while lying awake, and not to think of anything in particular or in a systematic way. In functional runs, stimuli were projected onto a screen at the end of the scanner, and participants viewed stimuli through a mirror mounted on the head coil. Participants responded with a magnet-compatible button box. For structural image acquisition, we used 3D-T1-turbo field echo sequence with the following parameters: sagittal acquisition with TR = 8.3 ms, TE = 3.3 ms, FOV = 198 × 220 mm^2^, voxel size = 0.77 × 0.86 × 1.0 mm^3^, 216 slices, flip angle = 12°, no gap.

### fMRI data analysis

#### Task fMRI analysis

Preprocessing and general linear model were conducted using Statistical Parametric Mapping 8 (SPM8 with MATLAB 2014a, Wellcome Department of Cognitive Neurology, London, U.K.). Slice-timing correction was conducted by resampling all slices relative to the middle slice (i.e., 17th slice) in temporal order. The fMRI data for each subject was realigned to the first volume for motion correction and only data sets with ≤ 2 mm maximal displacement during an entire scan were included in this study. Next, the functional images were coregistered to the T1-weighted image and spatially normalized to the Montreal Neurological Institute (MNI) template provided with SPM8, then resampled into 3 × 3 × 3 mm size voxels, followed by spatial smoothing using a Gaussian kernel with a full width at half maximum (FWHM) of 8 mm. A high-pass filter of 1/128 Hz was used to remove low-frequency noise, and temporal autocorrelation was done with an AR(1) + white noise model.

The general linear model (GLM) included 3 separate task regressors modeling activation during the 0-back, 1-back and 2-back blocks, and these regressors were convolved with canonical hemodynamic response function (HRF). Six motion parameters acquired during realignment and a session mean regressor were also included in the design matrix. Three beta images representing BOLD activity during each condition were generated for each individual participant, and these images were entered into the group-level analysis. For the 2^nd^ level analysis, 2(groups) x 3(conditions) full factorial ANOVA were conducted. The between-group factor consisted of “postpartum” and “control”, and the within-group factor consisted of “0-back”, “1-back” and “2-back”. All statistical analyses were corrected for multiple comparisons based on Monte Carlo simulation corresponding to an alpha level of *P* < 0.05^38^.

#### Resting-state fMRI analysis

An independent resting-state analysis was conducted to investigate intrinsic alterations of the postpartum group which were not affected by task-related activity. We conducted amplitude of low frequency fluctuations (ALFF) and fractional ALFF (fALFF) analyses to assess amplitude of intrinsic brain activity, and regional homogeneity (ReHo) analysis to assess regional connectivity of intrinsic brain activity using the toolboxes of Data Processing Assistant for Resting-state fMRI (DPARSF version 2.3, www.restfmri.net). ALFF/fALFF analyses demonstrated low frequency amplitudes which reflect spontaneous activation during the resting state^39^. In these analyses, data were preprocessed in an identical manner to that of the previous GLM analysis except that nuisance regression was excluded, and additional linear-trend removal and band-pass filtering (0.01–0.08 Hz) were conducted. Then, the time series for each voxel was converted to the frequency domain using a Fast Fourier Transform (FFT). The square root of the power spectrum was calculated and averaged across the low frequency range (0.01–0.08 Hz), yielding an ALFF value for each voxel. fALFF was obtained by computing the fraction of ALFF over the whole detectable frequency range for the given signal. The ALFF and fALFF maps of each participant were normalized to conduct the group-level two-sample t-test. ReHo analysis estimated voxel-based synchrony of time-series within a functional cluster during resting-state, which allowed us to examine regional functionality. The data were preprocessed with the same methods used in the ALFF/fALFF analysis, but smoothing was not performed in ReHo analysis to avoid inflating the results^40^. Then, Kendall’s coefficient of concordance (KCC) which represents regional similarity was calculated on the time series of a given voxel with those of its nearest 26 neighbors in a voxel-wise manner^41^. The resulting ReHo maps were also normalized to reduce variability across participants and underwent a two-sample t-test.

We used these three different resting-state analyses to identify neural alterations consistently found in both amplitude and connectivity aspects in the postpartum group during resting-state. Details for the resting-state analysis are described in the Supplementary information.

### Task-related network connectivity analysis

#### Network node selection

**Figure 6:**
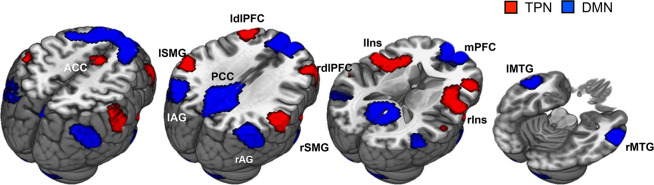
Six DMN and seven TPN nodes for the network connectivity analysis were derived from the resting-state functional connectivity analysis. Abbreviations: ACC, anterior cingulate cortex; lAG, rAG, bilateral angular gyrus; laIns, raIns, bilateral anterior insula; ldlPFC, rdlPFC, bilateral dorsolateral prefrontal cortex; lMTG, rMTG, bilateral medial temporal gyrus; lSMG, rSMG, bilateral supramarginal gyrus mPFC, medial prefrontal cortex; PCC, posterior cingulate cortex.

The DMN and TPN node selection was performed in the rsfMRI. First, the previously reported posterior cingulate cortex (PCC) seed [−2 −51 27] (Talairach coordinates) was selected as *a priori* ROI to generate functional connectivity maps^42^. The correlation coefficient map was constructed for each participant by calculating correlation between the time series of the 8 mm sphere PCC mask and that of each remaining voxel in the whole brain. The z-transformed functional connectivity (FC) maps were then averaged to generate a group-level DMN map. As the TPN is defined as regions showing anti-correlation with the DMN^12^, −1 weighted FC maps were used to create a TPN map. The nodes within each network were created as sufficiently large clusters (K > 68 voxels) after thresholding the DMN and TPN maps at Z > 0.35. Three clusters located in the cerebellum region were excluded from the DMN nodes, as considerable portions of the created clusters were positioned outside the brain. Six generated DMN nodes were located in the medial prefrontal cortex (mPFC), PCC, bilateral medial temporal gyrus (lMTG, rMTG) and bilateral angular gyrus (lAG, rAG), and seven TPN nodes were located in the dorsal anterior cingulate cortex (dACC), bilateral dorsolateral prefrontal cortex (ldlPFC, rdlPFC), bilateral anterior insula (laIns, raIns), and bilateral supramarginal gyrus (lSMG, rSMG) (Fig. 6).

#### Calculation of task-related network functional connectivity

The time series of each of the 13 nodes were extracted from preprocessed working memory task images (260 TR) for each participant. The time series of each voxel was detrended and normalized, and then averaged across voxels, yielding the time series of each node. Pair-wise cross correlation coefficients were calculated between each pair of the nodes by conducting a partial correlation, while controlling for motion artifacts, and the global mean and working memory load effect. Specifically, 3 cognitive load regressors for each condition (0-back, 1-back, 2-back) and 6 motion regressors that were convolved with HRF were regressed out. Then, the correlation coefficients were normalized by Fisher’s Z transform to increase normality. For each participant, within-DMN connectivity was assessed by averaging the correlation coefficients of each pair within DMN nodes, and within-TPN connectivity was assessed by averaging the correlation coefficients of each pair within TPN nodes. Inter-network connectivity was calculated by averaging the correlation coefficients of each pair between the DMN and TPN nodes. In order to compare within- and inter-network patterns between the postpartum and control groups, we carried out a two-sample t-test using each participant’s within-DMN, within-TPN, and inter-network connections.

#### Voxel-based morphometry analysis

To explore if there was any structural difference between the postpartum group and the control group and to find how the structural alterations were related to functional changes, we performed voxel-based morphometry analysis. Voxel-based morphometry analysis was conducted using SPM 12 (Wellcome Institute of Neurology, University College London, UK, http://www.fil.ion.ucl.ac.uk/spm/). T1-weighted images were segmented into gray matter, white matter, and cerebrospinal fluid by an image-intensity nonuniformity correction and a unified tissue-segmentation procedure. The segmented gray matter images were registered using a customized template by DARTEL algorithm (Wellcome Department of Imaging Neuroscience) and spatially normalized to standard MNI space. Then, the gray matter images were modulated using Jacobean determinants of the corresponding deformation filed for correcting volume changes. The resulting images were smoothed using an 8-mm FWHM Gaussian kernel.

The two-sample t-test was performed on gray matter images for group-level analysis, and we used an uncorrected threshold of P < 0.001, k = 100 for exploratory purposes.

### Clinical data analysis

Normally distributed clinical data were compared between the postpartum and control groups with the two-sample *t*-test. Other data were analyzed with the Mann-Whitney *U*-test.

Correlations between estradiol level, behavioral result, and network connectivity were examined. The Pearson correlation analysis was conducted if both values of the correlation pair were normally distributed, while the Spearman correlation analysis was conducted for correlation pairs with non-normal distributions.

Statistical analyses were performed with SPSS, Version 24.0 (IBM, Armonk, New York) and ﻿the statistical software R (version 3.5.1; R Foundation for Statistical Computing, Vienna, Austria). A two-tailed *P* < 0.05 was considered to indicate statistical significance.

## Supplementary information

Supplementary Information.

## Data Availability

The datasets analyzed during the current study are available from the corresponding author on reasonable request.

## References

[1] Casey P (2000). A longitudinal study of cognitive performance during pregnancy and new motherhood. Arch. Womens. Ment. Health.

[2] Casey P, Huntsdale C, Angus G, Janes C (1999). Memory in pregnancy. II: Implicit, incidental, explicit, semantic, short-term, working and prospective memory in primigravid, multigravid and postpartum women. J. Psychosom. Obstet. Gynaecol..

[3] Crawley RA, Dennison K, Carter C (2003). Cognition in pregnancy and the first year post-partum. Psychol. Psychother..

[4] Anderson MV, Rutherford MD (2012). Cognitive reorganization and protective mechanisms in pregnancy and the postpartum period. Evol. Psychol.

[5] de Groot RHM, Vuurman EFPM, Hornstra G (2006). & Jolles, J. Differences in cognitive performance during pregnancy and early motherhood. Psychol. Med.

[6] Henry JF, Sherwin BB (2012). Hormones and cognitive functioning during late pregnancy and postpartum: a longitudinal study. Behav. Neurosci..

[7] Henry JD, Rendell PG (2007). A review of the impact of pregnancy on memory function. J. Clin. Exp. Neuropsychol..

[8] Logan DM, Hill KR, Jones R, Holt-Lunstad J, Larson MJ (2014). How do memory and attention change with pregnancy and childbirth? A controlled longitudinal examination of neuropsychological functioning in pregnant and postpartum women. J. Clin. Exp. Neuropsychol..

[9] Baddeley A, Logie R, Bressi S, Sala S (1986). Della & al, et. Dementia and working memory. Q. J. Exp. Psychol. A Hum. Exp. Psychol..

[10] Ernst T, Chang L, Jovicich J, Ames N, Arnold S (2002). Abnormal brain activation on functional MRI in cognitively asymptomatic HIV patients. Neurology.

[11] Zimbelman JL (2007). fMRI detection of early neural dysfunction in preclinical Huntington’s disease. J. Int. Neuropsychol. Soc..

[12] Fox MD (2005). The human brain is intrinsically organized into dynamic, anticorrelated functional networks. Proc. Natl. Acad. Sci..

[13] Owen AM, McMillan KM, Laird AR, Bullmore E (2005). N-back working memory paradigm: A meta-analysis of normative functional neuroimaging studies. Hum. Brain Mapp..

[14] Sambataro F (2010). Age-related alterations in default mode network: impact on working memory performance. Neurobiol. Aging.

[15] Hampson M (2006). Brain connectivity related to working memory performance. J. Neurosci..

[16] Zou Q (2013). Intrinsic resting-state activity predicts working memory brain activation and behavioral performance. Hum. Brain Mapp..

[17] Hendrick V, Altshuler LL, Suri R (1998). Hormonal changes in the postpartum and implications for postpartum depression. Psychosomatics.

[18] Grigorova M, Sherwin BB, Tulandi T (2006). Effects of treatment with leuprolide acetate depot on working memory and executive functions in young premenopausal women. Psychoneuroendocrinology.

[19] Maki PM, Rich JB, Rosenbaum RS (2002). Implicit memory varies across the menstrual cycle: estrogen effects in young women. Neuropsychologia.

[20] Duff SJ, Hampson E (2000). A beneficial effect of estrogen on working memory in postmenopausal women taking hormone replacement therapy. Horm. Behav..

[21] Sherwin BB (1988). Estrogen and/or androgen replacement therapy and cognitive functioning in surgically menopausal women. Psychoneuroendocrinology.

[22] Crawley R, Grant S, Hinshaw KIM (2008). Cognitive changes in pregnancy: Mild decline or societal stereotype?. Appl. Cogn. Psychol..

[23] Rendell PG, Henry JD (2008). Prospective-memory functioning is affected during pregnancy and postpartum. J. Clin. Exp. Neuropsychol..

[24] Cuttler C, Graf P, Pawluski JL, Galea LAM (2011). Everyday life memory deficits in pregnant women. Can. J. Exp. Psychol. Can. Psychol. expérimentale.

[25] Clément F, Belleville S (2010). Compensation and disease severity on the memory-related activations in mild cognitive impairment. Biol. Psychiatry.

[26] Grady CL (2003). Evidence from functional neuroimaging of a compensatory prefrontal network in Alzheimer’s disease. J. Neurosci..

[27] Corriveau-Lecavalier N, Mellah S, Clément F, Belleville S (2019). Evidence of parietal hyperactivation in individuals with mild cognitive impairment who progressed to dementia: A longitudinal fMRI study. NeuroImage Clin..

[28] Hoekzema Elseline, Barba-Müller Erika, Pozzobon Cristina, Picado Marisol, Lucco Florencio, García-García David, Soliva Juan Carlos, Tobeña Adolf, Desco Manuel, Crone Eveline A, Ballesteros Agustín, Carmona Susanna, Vilarroya Oscar (2016). Pregnancy leads to long-lasting changes in human brain structure. Nature Neuroscience.

[29] Raichle ME, Snyder AZ (2007). A default mode of brain function: a brief history of an evolving idea. Neuroimage.

[30] Cole MW, Ito T, Bassett DS, Schultz DH (2016). Activity flow over resting-state networks shapes cognitive task activations. Nat. Neurosci..

[31] Joseph JE, Swearingen JE, Corbly CR, Curry TE, Kelly TH (2012). Influence of estradiol on functional brain organization for working memory. Neuroimage.

[32] Dumas JA, Kutz AM, Naylor MR, Johnson JV, Newhouse PA (2010). Increased memory load-related frontal activation after estradiol treatment in postmenopausal women. Horm. Behav..

[33] Beck AT, Ward CH, Mendelson M, Mock J, ERBAUGH J (1961). An inventory for measuring depression. Arch. Gen. Psychiatry.

[34] Cox JL, Holden JM, Sagovsky R (1987). Detection of postnatal depression. Development of the 10-item Edinburgh Postnatal Depression Scale. Br. J. psychiatry.

[35] Broadbent DE, Cooper PF, FitzGerald P, Parkes KR (1982). The cognitive failures questionnaire (CFQ) and its correlates. Br. J. Clin. Psychol..

[36] Yeom TH, Park YS, Oh KJ, Lee YH (1992). Korean version Wechsler adult intelligence scale. Seoul Korean Guid.

[37] Lee JH (2002). Development of the Korean Version of the Consortium to Establish a Registry for Alzheimer’s Disease Assessment Packet (CERAD-K) clinical and neuropsychological assessment batteries. Journals Gerontol. Ser. B Psychol. Sci. Soc. Sci.

[38] Slotnick SD, Moo LR, Segal JB, Hart J (2003). Distinct prefrontal cortex activity associated with item memory and source memory for visual shapes. Cogn. Brain Res..

[39] Yan. DPARSF: a MATLAB toolbox for “pipeline” data analysis of resting-state fMRI. *Front. Syst. Neurosci*. **4**, 1–7 (2010). 10.3389/fnsys.2010.00013 PMC2889691 20577591

[40] Zang Y, Jiang T, Lu Y, He Y, Tian L (2004). Regional homogeneity approach to fMRI data analysis. Neuroimage.

[41] Gibbons, J. D. & Kendall, M. G. *Rank correlation methods*. Edward Arnold (1990).

[42] Greicius MD, Krasnow B, Reiss AL, Menon V (2003). Functional connectivity in the resting brain: a network analysis of the default mode hypothesis. Proc. Natl. Acad. Sci..

