# Supplementary material for: Altered neural substrates within cognitive networks of postpartum women during working memory process and resting-state
Source: Sci Rep. 2020 Jun 4;10:9110. doi: 10.1038/s41598-020-66058-x (PMC7272423; doi:10.1038/s41598-020-66058-x)
Supplement: Supplementary file 1 — Supplementary Information. [file 41598_2020_66058_MOESM1_ESM.pdf]

Title

Altered neural substrates within cognitive networks of postpartum women during working memory process and resting-state

Authors

Yunjin Bak, Yoonjin Nah, Sanghoon Han, Seung-Koo Lee, Na-Young Shin*

Supplementary Information

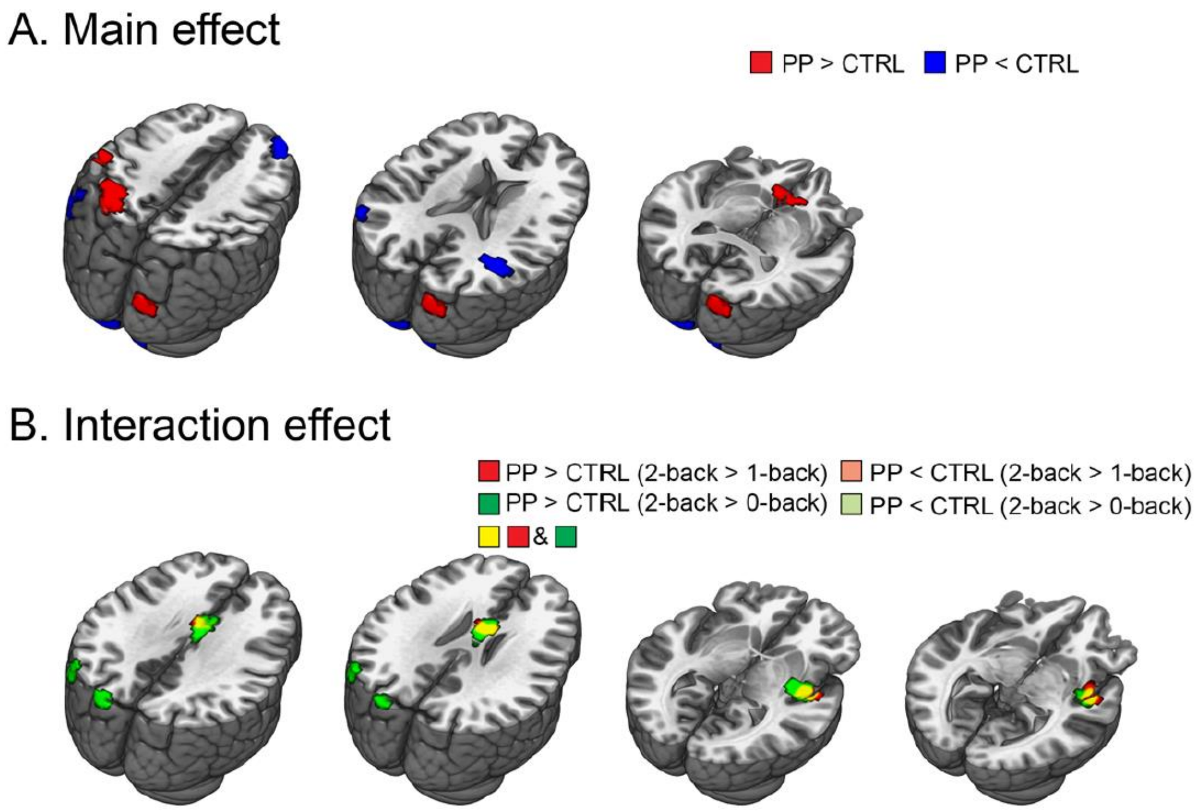

Supplementary Figure S1. n-back task-based fMRI activation analysis results after BDI, CFQ, and number of awakening were included as covariates. **A.** Main effects revealed by 2 (group: PP vs. CTRL) x 3 (task: 0-back vs. 1-back vs. 2-back) full factorial analysis. **B.** Interaction effects revealed by 2 (group: PP vs. CTRL) x 3 (task: 0-back vs. 1-back vs. 2-back) full factorial analysis. Abbreviations: BDI, Beck Depression Inventory; CFQ, Cognitive Failure Questionnaire; CTRL, control group, PP: postpartum group.
